# Non-Free Cutting Mechanism of Asymmetrical Nanogrooves Under Chip-Removal Interference in Amorphous Nickel Phosphorus

**DOI:** 10.3390/mi16091059

**Published:** 2025-09-18

**Authors:** Yupeng He, Yingzhao Cai, Minkun Huang, Benshuai Ruan, Peng Liu, Tianfeng Zhou

**Affiliations:** 1 School of Mechanical Engineering, Sichuan University, Chengdu 610065, China; heyp@scu.edu.cn (Y.H.); 15284185112@163.com (Y.C.); 18011524942@163.com (M.H.); 2 School of Mechanical Engineering, Beijing Institute of Technology, Beijing 100081, China; zhoutf@bit.edu.cn; 3 Chaofeng Micro-Nano Technology (Ningbo) Co., Ltd., Ningbo 315502, China; ruanbenshuai@cheerfulnano.com

**Keywords:** non-free cutting, asymmetrical nanogrooves, deformation, chip removal, shear interference

## Abstract

Asymmetrical nanogrooves are commonly employed as blazed gratings for precision measurement, optical communication, and optical sensing applications. Diamond cutting is a promising deterministic processing technology for nanogrooves with a triangular cross-section profile. Non-free cutting of nanogrooves makes it hard to suppress the cutting-caused deformation because of the low stiffness of nanogrooves. Focusing on the influence of non-free cutting on the deformation of asymmetrical nanogrooves, this paper systematically investigates the asymmetrical nanogroove cutting in amorphous nickel phosphorous material through mechanism revelation, simulation analysis, and experimental discussion. The materials removal mechanism by two side edges with different slopes in the non-free cutting is revealed according to the shear interference. According to the relative feed direction between tool and workpiece, two types of feed cases in the asymmetrical nanogrooves, named D1 and D2, respectively, are investigated by comparison in terms of deformation mechanism, nanogrooves topography, and nodal stress of tool edges. The extrusion by tool edges and the squeeze by the chip flow mainly influence the deformation of nanogrooves. In the D1 case, the horizontal component of squeeze by the chip flow towards the rear just-fabricated nanogroove, and the severely deformed nanogrooves are stacking together. On the contrary, in the D2 case, the flowing chip squeezes the front uncut materials, relieving the cutting-caused deformation, and asymmetrical nanogrooves have clear V-shaped cross-section profiles. It is proven that the D2 strategy is more suitable for asymmetrical nanogroove machining. The work in this paper will contribute to further understanding of non-free cutting and the processing technology of asymmetrical nanogrooves.

## 1. Introduction

Asymmetrical nanogroove features are V-shaped grooves with two side walls of different slopes, which are critical in applications such as optical diffraction gratings [1,2,3], microfluidic channels, anti-reflective surfaces [4], and bio-sensing devices [5,6]. As for the nanogrooves fabrication, achieving consistent geometry, low surface roughness, and defect-free features at the nanoscale is technically demanding. There are ultra-fast laser beams [7,8], plasma ICP etching [9], and diamond cutting [10,11] technologies suitable for fabricating complex-shaped structures with nanoscale features. However, fabricating V-shaped asymmetrical nanogrooves demands a promising deterministic manufacturing technology [12,13]. Among those, single-point diamond cutting offers exceptional precision [14,15], superior surface finish compared to conventional nanofabrication methods, since the cross-section of fabricated grooves is almost consistent with the shape of the tool tip [16,17,18]. Amorphous Nickel phosphorus (a-Ni-P) plating coated on a hard substrate is usually used as a mold material for high-precision injection molding and glass molding [19]. Therefore, nano-grooving on a-Ni-P workpiece has significant importance because of its wide application and superior micro–nano cutting performance [20,21]. In the micro/nano scale of cutting, the a-Ni-P materials exhibit superior plasticity [19]. Meanwhile, in the V-shaped nanogroove cutting process, cutting edges with different spatial angles for the tool tip is simultaneously involved in material removal. Therefore, V-shaped groove cutting of a-Ni-P is a special non-free cutting process, in which researchers found that the shear interference to materials and the chip-removal interference are the internal mechanisms that mainly influence the machining quality [22]. Correspondingly, the model of variable shear angle for describing the non-free cutting process is also established. It is found that the shear and chip-removal interference could cause the deformation of nanogrooves because the stiffness of nanogrooves is very small. In addition, researchers also found that the chip-removal interference could be properly suppressed to relieve the nanogrooves deformation by controlling the nominal rake angle of the V-shaped tool [23].

The existing research mainly focuses on symmetrical groove non-free cutting and the corresponding evolution law of cutting-caused deformation [22], in which the shear surface by the two side edges is symmetrical. However, for the asymmetrical nanogrooves non-free cutting, the position of shear interference and chip flow direction is asymmetrical and tilted to one side, causing the cutting-caused deformation to be influenced by the feed direction, chip formation behavior. Therefore, there is a lack of systematic understanding of the non-free cutting mechanism of asymmetric geometries, which will help in improving machining strategies.

Therefore, this paper proposes to investigate the non-free cutting mechanism of asymmetrical nanogrooves of a-Ni-P and the cutting-caused deformation. Firstly, based on the non-free cutting theory, the materials’ shear interference in the asymmetrical nanogrooves is analyzed to determine the materials shear surface, and then the mechanics analysis is conducted to reveal the internal cause of deformation of asymmetrical nanogrooves. Asymmetrical nanogrooves with 90° and 52° slopes are set as the object. According to the relative feed direction between tool and workpiece, two types of feed cases in the asymmetrical nanogrooves, named D1 and D2, respectively, are investigated by simulation and experiments in terms of deformation mechanism, nanogrooves topography, and nodal stress of tool edges. Finally, the deformation law of asymmetrical nanogrooves is further investigated and determined. Findings in this paper contribute to the precision manufacturing of non-symmetric nanostructures in high-value applications, such as optoelectronics, biomedical devices, etc.

## 2. Non-Free Cutting Mechanism of Asymmetrical Nanogrooves

### 2.1. Materials Shear Interference in the Cutting of Asymmetrical Nanogrooves

The V-shaped nanogroove cutting is a strong non-free cutting process; the materials shear effect by the two side edges interferes with each other, causing the extrusion effect by the tool edges to the nanogrooves amplified [22]. The flow direction of the chips is along the symmetrical line between the two side edges. The existing research only investigated the symmetrical nanogroove cutting in terms of the shear interference and cutting-caused deformation by the tool edge.

**Figure 1:**
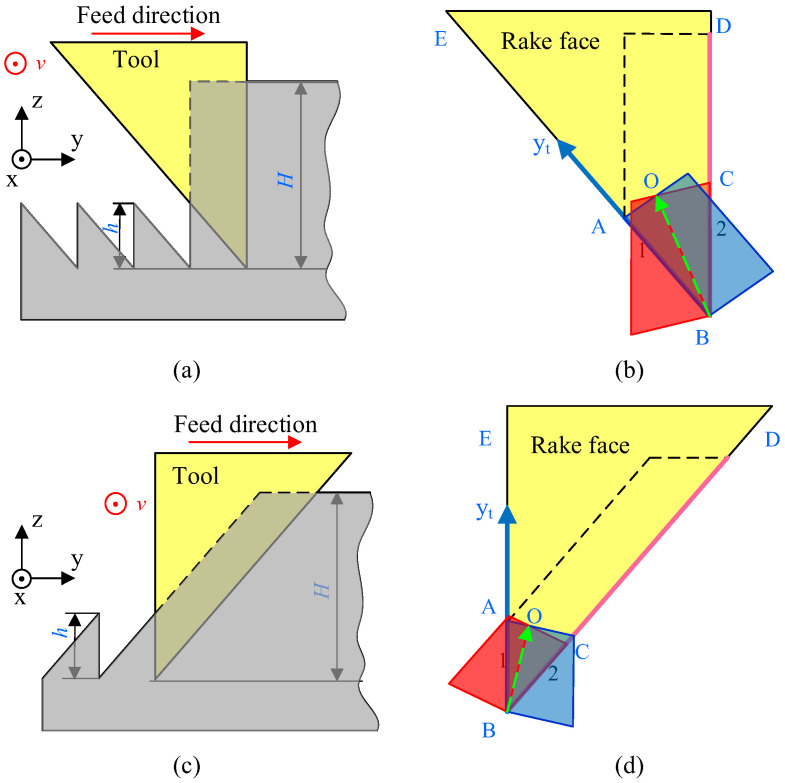
Illustration of non-free cutting of asymmetrical nanogrooves. (**a**) Cutting in case D1, in which the sloped 90° tool edge is front; (**b**) the schematic of interference of shear surfaces in D1 case; (**c**) cutting in case D2, in which the sloped 52° tool edge is front; (**d**) the schematic of interference of shear surfaces in D2 case.

However, for the asymmetrical groove cutting, the material shear interference is affected by the relative feed direction. As depicted in Figure 1, for the asymmetrical groove cutting, there are two types of relative feed directions, named D1 and D2, respectively. To study the influence of these two types of relative feed directions on the non-free cutting states, the feed direction is supposed towards right from left, and two cutting tools are used to fabricate asymmetrical nanogrooves. In this paper, the cutting tool with a nose angle of 38° is used to fabricate the asymmetrical groove, of which two side-slopes have slopes of 90° and 52°, respectively.

Figure 1a shows the D1 cutting circumstance, in which the sloped 90° tool edge (BC edge) is front, and the sloped 52° tool edge (AB edge) squeezes the being-cut nanogroove. According to the non-free cutting theory [22], the materials’ shear surfaces by the front and rear tool edges interfere in the materials and, therefore, the shear surface during nanogroove cutting is a 3D surface. Figure 1b shows the shear interference of the D1 for asymmetrical groove cutting. The front edge BC and rear edge AB cut the materials simultaneously, and the shear surface interferes in line BO. Meanwhile, the non-free cutting chip flows along the vector BO, which is tilted to the rear fabricated nanogrooves. Similarly, Figure 1c illustrates the D2 case for the asymmetrical groove cutting, in which the sloped 52° tool edge is front, and the sloped 90° tool edge squeezes the being-cut nanogroove. For the same reason, Figure 1d depicts the materials shear interference of the D2 case, showing that the interference line and chip flow direction BO are tilted to the front uncut materials.

### 2.2. Material Shear Surface During Asymmetrical Grooves

**Figure 2:**
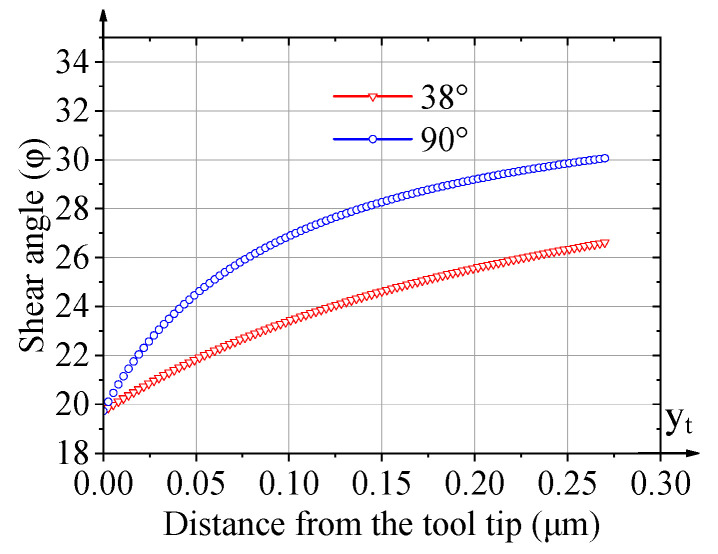
Comparison of local shear angles at tool edges with nose angle of 90° and 38°.

According to the existing non-free cutting theory, the shear angle is variable along the edge because of the material’s shear interference. Therefore, to calculate the 3D shear surface of the cutting of an asymmetrical groove, the shear angle at the tool edge should be calculated. And it can be decided according to the reference [21] by substituting the nose angle and the limitation value of the shear angle. Therefore, Figure 2 shows the trend of the calculated shear angle at the tool edge with tool nose angles of 90° and 38°. It could be noted that the tool nose angle of 38° makes the shear angle smaller because of the more severe material shear interference, compared with the nose angle of 90°. Therefore, during the non-free cutting process of nanogrooves, a smaller nose angle causes more significant chip removal interference between the two tool edges, further deteriorating the cutting state.

**Figure 3:**
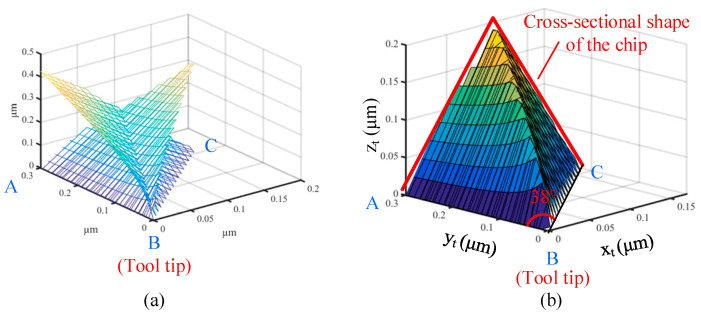
The shear surface of the non-free cutting of 38°-angled nanogrooves. (**a**) Shear surface of front and rear edges, (**b**) the whole 3D shear surface and chip cross-section of non-free cutting.

The respective shear surface by the front and rear edges is calculated and depicted based on the obtained variable shear angle, as shown in Figure 3a. Therefore, the whole shear surface can be obtained as Figure 3b, in which the boundary of the shear surface represents the cross-sectional shape of the chip. The 3D shear surface continuously forms in the materials and flows on the rake face, eventually generating the chip. By comparing the shear surface by tools with a nose angle of 38° with that of 90° in reference [22], the shear surface of the 38° V-shaped tool is steeper based on the presentation with the same scale. Therefore, the chip flow is more difficult during the cutting of 38°-angled nanogrooves.

### 2.3. Mechanics Analysis of Asymmetrical Nanogroove Cutting

**Figure 4:**
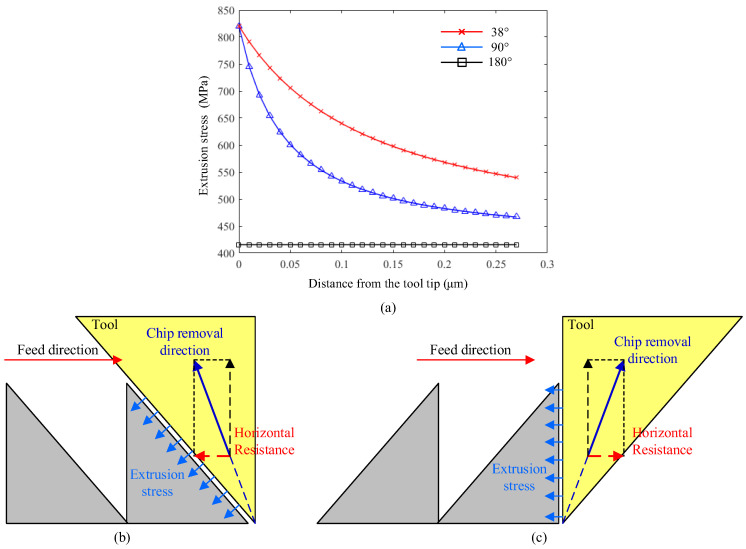
Mechanical analysis for asymmetrical nanogroove cutting; (**a**) extrusion stress distribution *σ* along the tool edge BA with nose angles of 90°, 38°, and 180°, schematic diagram of extrusion by the tool edge and the squeeze by flowing chip in (**b**) D1 case and (**c**) D2.

The extrusion stress by the rear edge BA to the nanogroove will cause the deformation of the nanogroove since its stiffness is very low. Based on the mechanical model reported by the reference, the extrusion stress *σ* and its distribution along the tool edge during 38°-angled nanogroove cutting are calculated and graphed in Figure 4a. Meanwhile, the results of the tool nose angles of 90° and 180° are exhibited to make the comparison. It shows that the decrease in nose angle obviously enhances the extrusion stress acting on the nanogrooves’ slope due to the more severe non-free cutting state. It is shown that extrusion stress becomes about two times larger at the area closer to the tool tip because of the higher degree of non-free cutting at the bottom area. Therefore, the valley area of the nanogroove suffers the highest squeeze stress from the tool edge. Figure 4b,c illustrate the extrusion stress from the rear edge to the nanogroove in two cases of cutting. Therefore, the extrusion stress actually acts on different sides of the triangle of the nanogroove in different cases, which influences the cutting-caused deformation of the nanogroove in terms of the different stiffness of the nanogroove.

In addition, there is a more important factor in different cases that greatly affects the deformation of asymmetrical nanogrooves. During the flow of the generated shear surface and chip, there is flow resistance in the opposite direction of flow. Therefore, the flowing chip will squeeze the materials along the chip flow direction. As shown in Figure 4b, in the D1 case of asymmetrical nanogroove cutting, the chip flow resistance has a horizontal component, which makes the chip squeeze the just-fabricated nanogroove towards the rear. And the nanogroove simultaneously suffers both the extrusion of the tool edge and the squeeze of the chip. For the D2 case, the horizontal resistance makes the chip squeeze the front uncut materials; therefore, the nanogrooves in the D2 case mainly suffer from the extrusion stress by the tool edge.

## 3. FEM Simulation of Asymmetrical Nanogroove Cutting

### 3.1. Material Settings and Simulation Methodology

As analyzed above, the cutting-caused deformation of the nanogroove is related to the stiffness, extrusion stress by the tool edge, and the squeeze of chip removal, of which results are investigated by the finite element method via Abaqus. In the Abaqus modeling and setting, the Johnson–Cook material constitutive model is utilized to resolve the large plastic deformation circumstances. In this simulation, the modeling method and material properties of the a-Ni-P workpiece and diamond tool are set the same as the reference [22]. A groove is preset on the rear side of the current groove, and monitor nodes are defined on the rear slope of the nanogroove to quantitatively analyze the deformation of cutting-caused deformation. The cutting speed is 9.4 m/s. Nodes on two side edges of the tool are extracted to analyze the degree of non-free cutting. Similar to the reference, only the elastic attribute is added to the diamond tool to proportionally represent the non-free cutting degree.

### 3.2. Simulation Result of Cutting-Caused Deformation of Asymmetrical Nanogrooves

**Figure 5:**
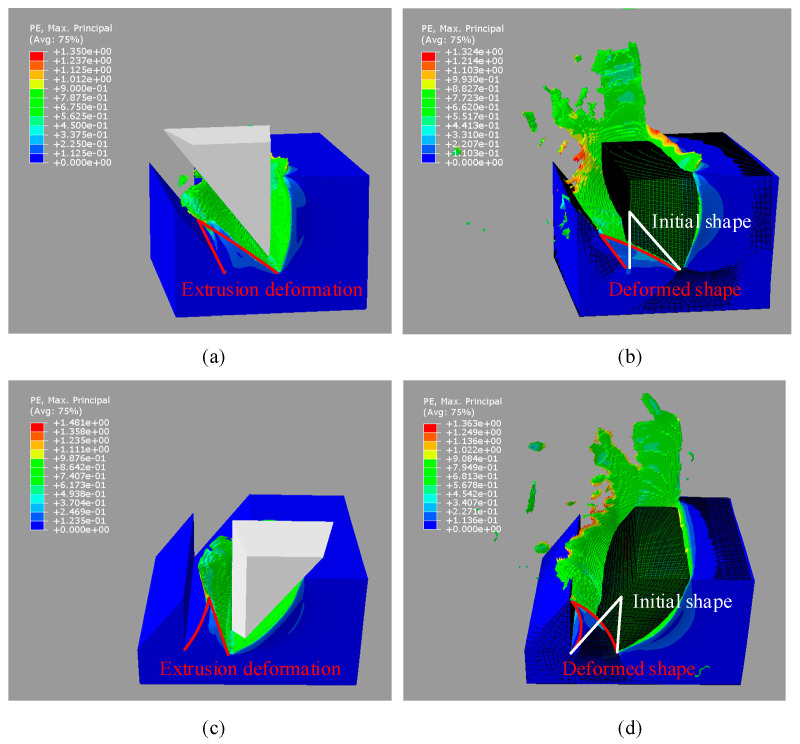
Simulation of asymmetrical nanogroove cutting by Abaqus. (**a**) The cutting-caused deformation of the asymmetrical nanogroove and (**b**) the comparison of the ideal shape and the deformed shape of the groove in case D1. (**c**) The cutting-caused deformation of the asymmetrical nanogroove and (**d**) the comparison of the ideal shape and the deformed shape of the groove in case D2.

**Figure 6:**
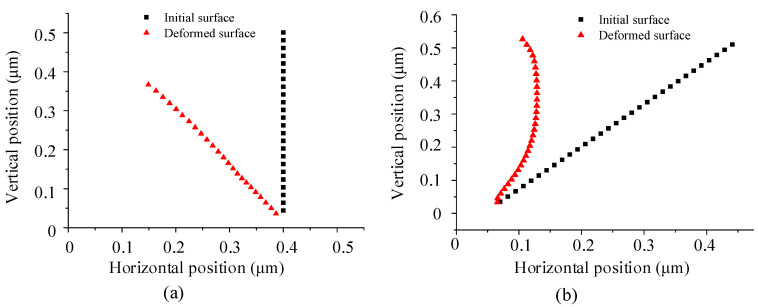
The position of monitor nodes on the rear slope of the groove before and after cutting in case (**a**) D1 and (**b**) case D2.

Figure 5a shows the cloud map of the asymmetrical nanogrooves cut by the tool with a nose angle of 38° in case D1. Figure 5b shows the comparison of the initial shape of the material before cutting and the deformed shape of the groove after cutting. Figure 6a shows the position of the rear slope of the groove before and after cutting; the initial surface of the rear slope is a vertical plane. The groove is severely deformed towards the rear, causing a large forward shift in the relative cutting line of the rear tool edge. In addition, a large amount of material is pushed to the rear side, forming a residue and stacking with the rear groove. At the same depth-of-cut (DoC), the deformation is smaller in D2 than that in D1, as shown in Figure 5c,d and Figure 6b. The initial angle of the rear slope of the nanogroove is 52°, and the cutting causes the bending deformation towards the rear. And a slight material residue is accumulated at the top of the groove after cutting. It should be noted that the bottom area of the nanogroove in the D2 case does not change much. A comparison of the two cutting cases indicates that the deformation of the asymmetrical nanogroove depends primarily on the structural stiffness and the biased direction of the chip flow. The nanogroove deformation is caused by the extrusion of the tool edge and the squeeze of chip flow. During the cutting of the same groove, it is also found that the deformation of the material at subsequent positions causes the previously machined surface to bend backward because the material is continuous. Therefore, the final cut-off surface is not consistent with the tool edge but is bent.

**Figure 7:**
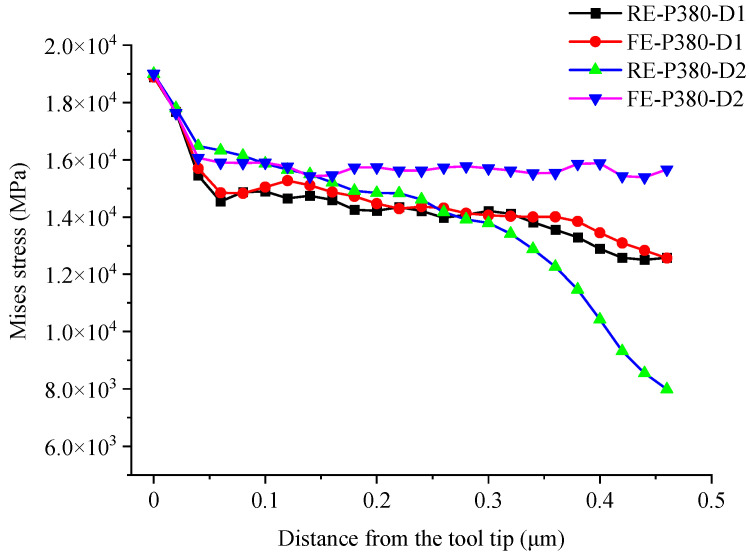
Nodal stress of the two side edges in cases D1 and D2.

Figure 7 shows the nodal stress of the tool edges on both sides in cases D1 and D2. In case D1, the nodal stress on both sides is symmetric. However, in case D2, the nodal stress of the rear tool edge decreases more sharply along the tool edge starting from the tool tip. Therefore, the deformation characteristics and the interaction mechanism between the tool and the material differ significantly between the two cases. According to the deformed shape of the nanogroove (Figure 6a), in case D1, the material is stacked with the rear groove because of the extrusion by the rear edge and the squeeze of the flowing chip, which provides support and increases stiffness. Therefore, the low stiffness of the nanogroove in D1case does not affect the nodal stress, which is only related to the degree of chip removal interference. However, in case D2, the rear material is not supported after the deformation; therefore, the nodal stress decreases substantially as the deformation increases.

## 4. Experiment of Asymmetrical Nanogroove Cutting

### 4.1. High-Speed Fly Cutting of Nanogrooves

**Figure 8:**
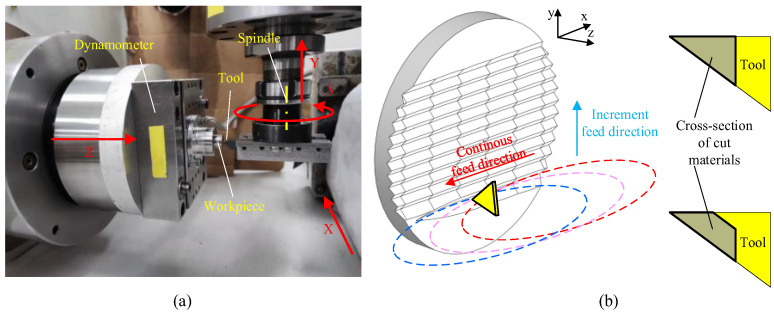
Experimental method for asymmetrical nanogroove cutting; (**a**) experimental setup and (**b**) the properties of the groove cutting process.

The speed of the translation servo axis usually cannot reach more than several meters per minute for a small ultra-precision machine. The fly cutting method is used to generate a high-speed linear cutting speed to fabricate nanogrooves. The schematic of radial-feed fly cutting used in this paper is shown in Figure 8, showing that one groove is fabricated by one or many rotations of the flying cutter. Therefore, the groove length is decided by the feed distance along the direction perpendicular to the rotating axis. And the groove period is controlled by the horizontal increment federate. Ultimately, the asymmetrical nanogroove cutting experiments are conducted on the ultra-precision machine (ULG-100, Toshiba, Tokyo, Japan). And a new V-shaped SCD tool with nose angle of 38° is used, of which edge radius is about 20–30 nm according to the data provided by the supplier.

It should be noted that feed speed perpendicular to the spindle does not influence the groove length but decides the distance between adjacent revolutions of the flying cutter. Therefore, when the spindle speed is fixed, the bigger feed speed makes most of the single nanogroove by one-step cut of the cutter, as shown in Figure 8b, which agrees with the analysis model of this paper.

The rotation radius *R* is 30 mm, and the rotation speed is fixed at 3000 r/min. Thus, the cutting speed of the tool tip is 9.4 m/s, which is consistent with the simulation setting. For all the nanogroove experiments, the feed speed perpendicular to the spindle axis is set to 100 mm/min. Groove periods of 380 nm, 1000 nm, 2000 nm, and 5000 nm are obtained by controlling the increment feed rate, respectively. A tool with a nose angle of 38° is used to create asymmetrical grooves, being mounted with 90° for the rear-side tool edge and 52° for the front-side tool edge. The deformation of the grooves with different periods is investigated in terms of the relative feed direction.

### 4.2. Deformation Analysis of Asymmetrical Nanogrooves

**Figure 9:**
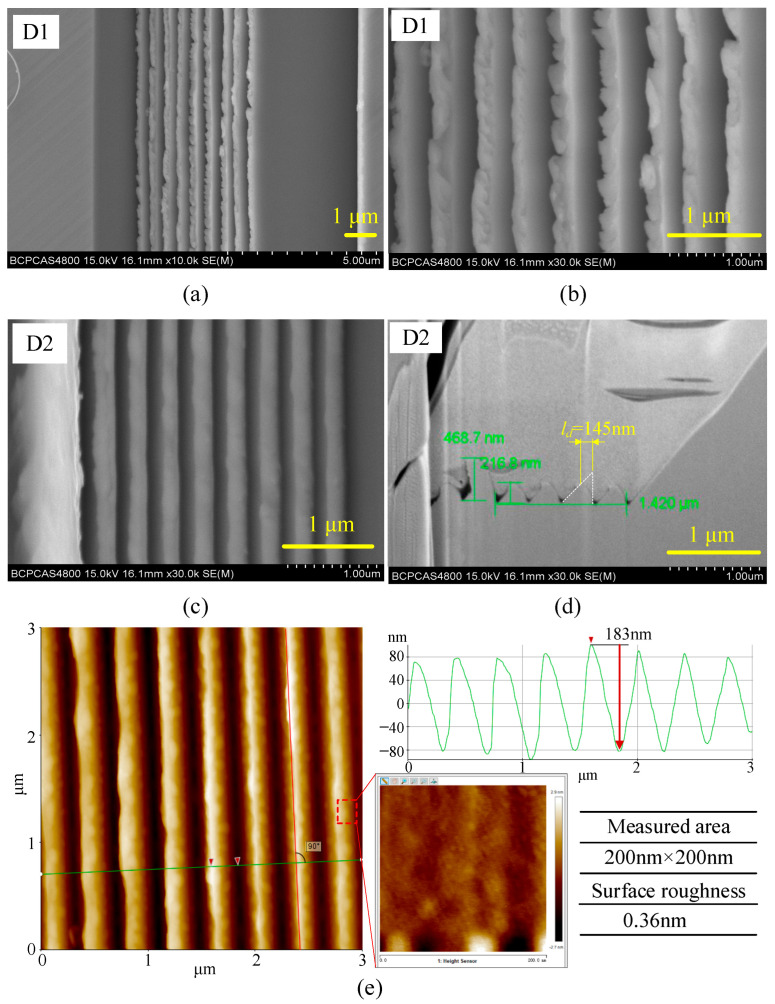
(**a**) The deformation and stack of the machined grooves, and (**b**) the detail at the top ridge of the deformed grooves machined in the D1 case. (**c**) Topography and (**d**) V-shaped cross-section of the machined grooves in the D2 case. (**e**) The measured cross-section profile and surface roughness by AFM for nanogrooves machined in the D2 case; and red arrows indicate the locations for measuring the size of the machined groove.

The machined asymmetrical nanogrooves with a period of 380 nm are shown in Figure 9. In the cutting of the D1 case, a few chips are produced. Consistent with the simulation results, the grooves exhibit severe deformation and are stacking together with the rear grooves, as shown in Figure 9a. The top ridge of the grooves is shown in detail in Figure 9b. The torn material and intermittent burrs caused by the cutting of the rear tool edge are observed, showing typical cutting features of plastic materials. Therefore, in the cutting of the D1 case, the groove is strongly deformed, and material flow occurs due to extrusion by the rear-side tool edge and the squeeze by the flowing chip. Thus, the relative cutting line of the rear-side tool edge in the material has shifted forward substantially. That is to say, in the cutting process, after being cut off by the front-side tool edge, only a small portion of the material is cut off by the rear-side tool edge. The rest is extruded backward to form a residue and stack with the other grooves. Since the severely deformed nanogrooves are stacking together, the deformation amount in D1 case could be regarded as 380 nm (equal to the groove period). As for the asymmetrical nanogrooves machined in the D2 case (Figure 9c), the generated grooves have clear V-shaped cross-section, indicating that the deformation of the groove and the material residue on the top ridge of the grooves are smaller than those in case D1. As analyzed in Section 2.3, in case D2, the chip flow direction is tilted to the front uncut materials, and the squeezed surface of the nanogrooves is a vertical surface, of which the stiffness is higher than that in case D1. The cross-section of the grooves in Figure 9d shows the details of the bending deformation in case D2. The cut-off surface is a bent surface, which is consistent with the simulation results. Because of the bending deformation, the height of machined nanogrooves is measured to be about 217 nm (including the measurement error), of which ideal value is 486 nm according to geometrical calculations. In Figure 9d, the white dotted lines profile the ideal V-shape cross-section of asymmetrical groove, which helps to quantitatively measure the deformation amount by measuring the deviation between the top vertexes of machined and ideal nanogrooves. The result shows that there is about 145 nm deformation amount for the asymmetrical nanogrooves machined in D2 case. Therefore, according to the characterization results of deformation, it is demonstrated that D2 strategy is more suitable for asymmetrical nanogroove machining.

To quantitatively characterize the machining quality of the asymmetrical nanogrooves in case D2, the atomic force microscope (AFM) measurement is conducted, as shown in Figure 9e. The nanogrooves height is measured to be 183 nm, lower than that measured from Figure 9d, which may be attributed to the probe being unable to fully contact the valley of the nanogrooves. In addition, an area with a size of 200 nm × 200 nm on the side slope of machined nanogroove is measured for surface roughness, which shows the Sa is 0.36 nm.

**Figure 10:**
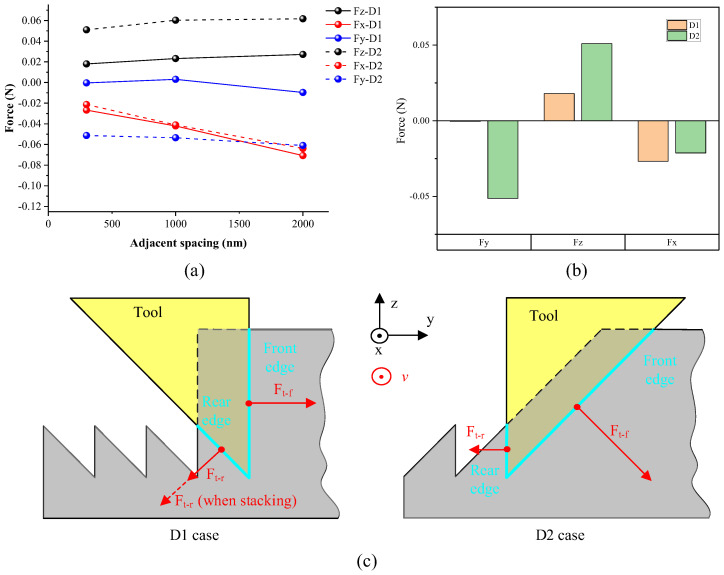
Cutting force result and analysis of asymmetrical nanogrooves of D1 and D2 cases. (**a**) Cutting force of nanogrooves with periods of 380 nm, 1000 nm, and 2000 nm; (**b**) cutting force comparison between D1 and D2 cases for nanogrooves with spacing of 380 nm; (**c**) analysis of thrust force on both front and rear edges in D1 and D2 cases.

The cutting force data in three directions is recorded during asymmetrical nanogroove cutting for both D1 and D2 cases, as graphed in Figure 10a, for nanogrooves with spacing of 380 nm, 1000 nm, and 2000 nm. It is shown that the difference in cutting force between D1 and D2 cases presents the same trend for different nanogrooves spacing. To analyze the difference in cutting force between D1 and D2 cases, the comparison between D1 and D2 cases is conducted for the nanogrooves with a spacing of 380 nm, as shown in Figure 10b. The deformation of asymmetrical nanogrooves during cutting has a large influence on the cutting force. In the D1 case, the horizontal resultant force (F_y_) of two tool edges is close to 0, indicating the horizontal component of cutting force of the rear edge is close to that of the front edge. Figure 10c illustrates the force vectors generated by tool edges, in which the thrust force of both front and rear tool edges is focused because it mainly leads to the cutting-caused deformation of nanogrooves. In the cutting process, the actual depth-of-cut must be much larger than the nanogroove height because of the tool setting error and nominal depth-of-cut. Therefore, it causes additional support for the rear-side nanogrooves after the deformation and stacking, so there is almost a similar horizontal force component on the rear and front tool edges. In the cutting of the D2 case, because the deformation of the nanogroove is smaller, these nanogrooves are isolated in space. Hencs, there is no additional physical support to the rear slope of the nanogroove, and the horizontal resultant force towards the rear. As illustrated in Figure 10c, the resultant force in the DoC direction is only from the 52°-sloped tool edge, so its value is larger in D2 than that in D1 since the 52°-sloped tool edge involved in the cutting is longer in the D2 case. According to the cutting force data, in the cutting of asymmetrical nanogrooves, the D2 cutting case, which helps to relieve the cutting-caused deformation, generates a larger cutting force in the feed direction. Therefore, it demonstrates that macroscopic cutting force data may underrepresent edge-specific interactions under non-free cutting. Because of this, edge-resolved sensing would be required to quantify forces per tool edge in the future.

**Figure 11:**
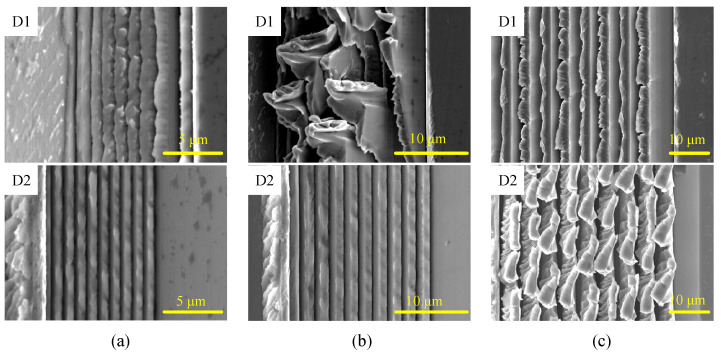
Machined grooves with large periods using asymmetrical cutting tools. (**a**) 1000 nm, (**b**) 2000 nm, and (**c**) 5000 nm.

As analyzed above, in different cutting cases, the relative increment feed direction is opposite, in which the deformation of the asymmetrical nanogrooves is significantly affected. The experimental results find that the deformation of nanogrooves shows a significant trend with the change in the groove period, as shown in Figure 11. For the D1 case of cutting, the asymmetrical nanogrooves with a period of 1000 nm are stacking and have a large deformation. During the cutting of asymmetrical grooves with a period of 2000 nm, no material is removed by the rear-side tool edge after the material has been cut off by the front-side tool edge. Therefore, the residue material accumulates and is stacked with the rear-side grooves. For grooves with a period of 5000 nm, because of their higher structural stiffness, part of the material is cut off and removed when cutting in the D1 case, but there is still some material residue left and tilted to the rear side on the top ridge of the grooves because the stiffness of the groove ridge is very small.

On the contrary, machined grooves with a period ranging from 380 nm to 2000 nm have higher quality by the cutting of case D2, as shown in Figure 10. Thus, the D2 strategy is more suitable for asymmetrical nanogroove machining. It should be noted that the removal amount by the tool edge increases with the increase in groove period, making it more difficult to remove the chip and increasing the chip-removal resistance. Therefore, for the cutting of nanogrooves with a period of 5000 nm in case D2, the top area of the nanogroove is deformed, and chips and material residue may be formed on the top ridge. In Figure 11c, the substantial uncut chip forms on the ridge of the grooves with a period of 5000 nm, suggesting that the removal amount in the one-step cutting process of asymmetrical nanogrooves of a-Ni-P material should be kept within 2000 nm, even in the case of D2.

## 5. Conclusions

In this paper, the non-free cutting of asymmetrical nanogrooves is systematically investigated from theory, simulation, and experiment, and therefore, new insights about the cutting-caused deformation of asymmetrical nanogrooves are formed in terms of mechanism, evolution law, and machining process. The main conclusions are as follows:

1. The analysis of shear interference and chip flow of non-free cutting of asymmetrical nanogrooves shows that extrusion by tool edges and squeezing by the chip flow mainly influence the deformation of nanogrooves. In 38° V-shaped nanogroove cutting, the strong degree of non-free cutting increases the extrusion stress. The flow resistance of the chip squeezes the low-stiffness nanogrooves, prone to exacerbating the deformation of nanogrooves.
2. The machining quality of asymmetrical nanogrooves is reliant on the relative feed direction between the tool and workpiece. Therefore, the asymmetrical nanogroove cutting is typed into two cases, D1 and D2, respectively. In the D1 case, the horizontal component of squeezing by the chip flow towards the just-fabricated nanogroove. On the contrary, in the D2 case, the flowing chip squeezes the front uncut materials, relieving the cutting-caused deformation.
3. Results of both simulation and experiment verify that the groove bending deformation in the D2 case is substantially suppressed compared with that in the D1 case. In the cutting of the D1 case, the severely deformed nanogrooves are stacked together. The analysis of cutting force finds that the macroscopic total force may underrepresent edge-specific interactions under non-free cutting. Because of this, edge-resolved sensing would be required to quantify forces per tool edge in the future.
4. D2 strategy is more suitable for asymmetrical nanogroove machining. However, the removal amount by the tool edge increases with the increase in groove period, making it more difficult to remove the chip and increasing the chip-removal resistance. Therefore, the top area of the nanogroove with a large period is deformed in the one-step cut process, and chips and material residue may be formed on the top ridge. It is suggested that the removal amount in the one-step cutting process of asymmetrical nanogrooves of a-Ni-P material should be kept within 2000 nm.

## Data Availability

The raw data supporting the conclusions of this article will be made available by the authors on request.
